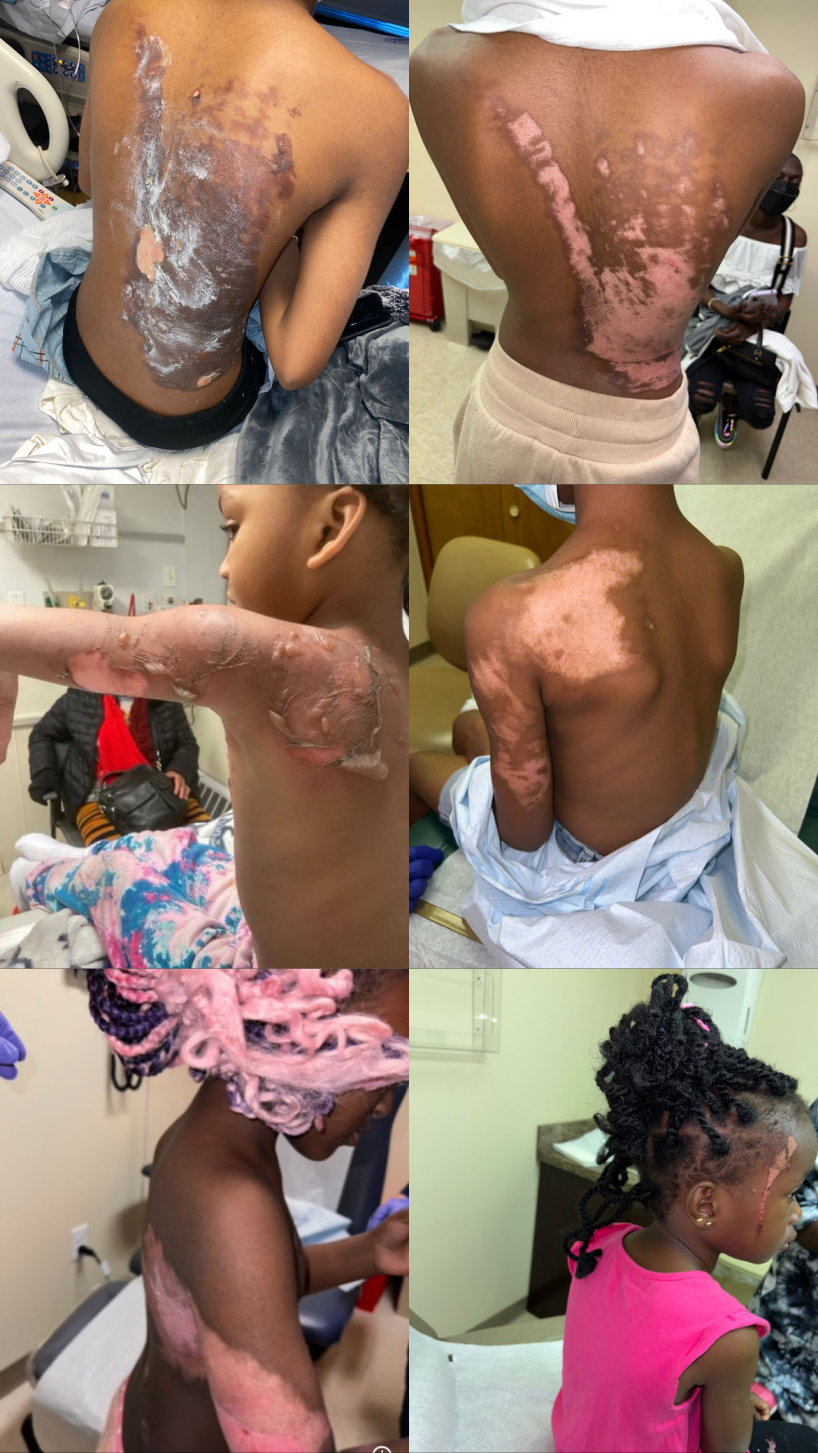# 553 Scald Burns in African American Girls and Women Associated with Hair Braiding

**DOI:** 10.1093/jbcr/iraf019.182

**Published:** 2025-04-01

**Authors:** Michael Cooper, Kelli Gills, Kimlyn Long, Diana Macaulay, David Lavallee, Nayana Parekh, Johnna Diouf

**Affiliations:** Staten Island University Hospital, Northwell Health; Northwell Health; Northwell Health; Northwell Health; Northwell Health; Northwell Health; Touro

## Abstract

**Introduction:**

Hair braids are a common hair style among African American girls and women. The ends of added synthetic hair are dipped in hot water containers placed behind the back to prevent unraveling. Second- and third-degree scald burns occur due to spilled hot water or contact with hot wet braids.

**Methods:**

Institutional Review Board approval was obtained. A retrospective study from April 2022 to February 2024. of nine patients with scald burns associated with hair braiding. The inpatient and outpatient electronic medical records were reviewed.

**Results:**

100% of patients were female and African American. There were 6 pediatric patients and 3 adult patients. The average age of pediatric patients was 8.5 years, and ranged from 3 to 17 years (3,6,7,7,11, 17). The adult ages were 24, 26, and 47, with an average age of 32 years. The percent Total Body Surface Area (%TBSA) ranged from 1% to 8% (1%, 2%, 3%, 4%, 5%, 5%, 6%, 7%, 8%), with an average of 4.5% TBSA. All pediatric burns occurred at home, and all adult burns occurred at hair salons. One adult was a client, and 2 adults were hairstylist. One hairstylist was burned while transporting aa kettle of hot water, and one hairstylist was assaulted with hot water by a disgruntled client’s mother. Six patients were admitted (5 pediatric and 1 adult). Three patients were treated as outpatients (1 pediatric patient and 2 adults). One pediatric patient’s burns were isolated to her face. Eight patients were burned to multiple body part. The back was the most commonly burned (4 patients or 44.4%). Buttocks (2), chest (3), bilateral corneas (1), bilateral hands (1), flank (1), neck (1), ear (1), foot (1), arm (2), thigh (1), and shoulder (1) were also injured. Seven patients had second degree burns, and 2 pediatric. patients and second- and third-degree burns. Hypopigmentation was documented in 100% of patients.

**Conclusions:**

Hair braids are common and ubiquitous among African American girls and women, with synthetic hair often added. However, scald burns may occur due to dipping the hair in hot water, especially burns to the back. Patients may be hospitalized, miss school or work, have discolored skin, and develop burn scar keloids. Pediatrics patients are at high risk of scald burns associated hair braiding. They are treated at home by caregivers who are unaware that children often become frightened by hot water behind them, and either tip over the water or pull the hot wet hair onto themselves. This is also the first report of hairstylist scald burns associated with hair braids. Hairstylist should be aware of the risks of scald burns to client and self when transporting containers of hot water and should be discard hot water after use to prevent the possibility of assault.

**Applicability of Research to Practice:**

More awareness and education is needed for parents, caregivers, and hair stylist to prevent scald burns associated with hair braiding. In addition, a protective garment should be encouraged to protect the back and other body parts from scald burns associated with hair braids.

**Funding for the Study:**

N/A